# A Novel Role for RARα Agonists as Apolipoprotein CIII Inhibitors Identified from High Throughput Screening

**DOI:** 10.1038/s41598-017-05163-w

**Published:** 2017-07-19

**Authors:** Sang Jun Lee, Madhupriya Mahankali, Abdallah Bitar, Huafei Zou, Elizabeth Chao, Hung Nguyen, Jose Gonzalez, Dawna Caballero, Mitch Hull, Danling Wang, Peter G. Schultz, Weijun Shen

**Affiliations:** 1 California Institute for Biomedical Research (Calibr), 11119 North Torrey Pines Rd, Suite 100, La Jolla, CA 92307 USA; 2 Department of Chemistry, the Scripps Research Institute, 10550 North Torrey Pines Rd La Jolla, La Jolla, CA 92037 USA; 3 Scripps Translational Science Institute, 3344N Torrey Pines Ct, La Jolla, CA 92037 USA

## Abstract

Elevated triglyceride (TG) levels are well-correlated with the risk for cardiovascular disease (CVD). Apolipoprotein CIII (ApoC-III) is a key regulator of plasma TG levels through regulation of lipolysis and lipid synthesis. To identify novel regulators of TG levels, we carried out a high throughput screen (HTS) using an ApoC-III homogenous time resolved fluorescence (HTRF) assay. We identified several retinoic acid receptor (RAR) agonists that reduced secreted ApoC-III levels in human hepatic cell lines. The RARα specific agonist AM580 inhibited secreted ApoC-III by >80% in Hep3B cells with an EC_50_ ~2.9 nM. In high-fat diet induced fatty-liver mice, AM580 reduced ApoC-III levels in liver as well as in plasma (~60%). In addition, AM580 treatment effectively reduced body weight, hepatic and plasma TG, and total cholesterol (TC) levels. Mechanistically, AM580 suppresses ApoC-III synthesis by downregulation of HNF4α and upregulation of SHP1 expression. Collectively, these studies suggest that an RARα specific agonist may afford a new strategy for lipid-lowering and CVD risk reduction.

## Introduction

Apolipoprotein CIII (ApoC-III), a component of very low density lipoproteins (VLDL) and high density lipoproteins (HDL), is a 79-aa glycoprotein synthesized primarily in the liver and, to a lesser extent, by the intestines^1, 2^. Recent studies in rodent and human subjects have validated the role of ApoC-III as a key regulator of plasma triglyceride levels and potential risk for CVD^3–10^. Increased expression of ApoC-III is associated with severe hypertriglyceridemia in rodents (8), and also a characteristic feature of patients with hypertriglyceridemia^3^. Conversely, the loss-of-function mutation of ApoC-III in humans leads to decreased TG levels and reduced incidence of CVD^5, 10^; individuals lacking ApoC-III have low triglyceride-rich lipoproteins (TRL) levels coupled with highly efficient lipolysis of triglycerides^11^. ApoC-III homozygote knockout mice display hypotriglyceridemia and protection from postprandial hypertriglyceridemia^7^. It has been shown that ApoC-III induces alterations in serum TG levels by both extracellular and intracellular mechanisms. The extracellular activity of ApoC-III increases plasma TG levels by reducing the activity of lipoprotein lipase to hydrolyze triglyceride-rich lipoproteins (TRL)^11^ and by reducing the hepatic uptake of TRL^12–14^. The intracellular activity of ApoC-III promotes TG synthesis, VLDL assembly and VLDL secretion^15–17^.

Epidemiological and population-based research has also suggested another potential role for ApoC-III in CVD risk management^18^. In a *post-hoc* analysis from the Cholesterol and Recurrent Events (CARE) trial, a randomized placebo-controlled trial of pravastatin for secondary prevention of cardiovascular related events in patients with persistently elevated LDL concentrations, plasma ApoC-III levels were strong, independent predictors of cardiovascular events (RR 2.3, *P* = 0.04). In fact, ApoC-III levels were better and stronger predictors than plasma triglycerides levels (RR 1.3, *P* = 0.6). This is further substantiated by the American Heart Association (AHA) scientific statement wherein they stated that TGs alone are not pro-atherogenic unless they are associated with ApoC-III^19^.

Taken together, these studies suggest that a novel therapeutic approach to managing hypertriglyceridemia and CVD risk is the regulation of ApoC-III levels. Although it has only been studied in familial chylomicronemia syndrome (defective catalysis in LPL leading to elevated ApoC-III and TG levels), oligonucleotide antisense technology has yielded an inhibitor to ApoC-III messenger RNA, ISIS 304801, which has reduced ApoC-III serum levels by at least 71% among three patients^20^. The efficacy of several small molecule therapeutics like fenofibrates and statins also has been attributed in part through their indirect effect on plasma ApoC-III levels via PPARα activation^21–25^, although their mechanism of action is not fully understood. Large-scale screening of small molecule libraries directly targeting ApoC-III production, processing and secretion could potentially provide a novel therapeutic option to be added to the armamentarium of lipid-lowering agents for CVD risk-reduction. To this end, we have developed a novel cell-based homogenous time resolved fluorescence (HTRF) assay for secreted ApoC-III, conducted a high throughput screen of a library of a million small molecules, and identified several novel molecules and known drugs that affect ApoC-III levels. Among these are retinoic acid receptor (RAR) agonists which we chose to investigate in detail. AM580, an RARα specific agonist, inhibited ApoC-III mRNA and protein levels *in vitro* in hepatic cell lines as well as *in vivo* in mouse models. Oral dosing of AM580 in diet-induced fatty liver mice reduced liver and plasma ApoC-III levels, as well as body weight, total cholesterol (TC) and TG levels through inhibition of HNF4α and subsequent up-regulation of SHP1.

## Results

### ApoC-III uHTS assay development and optimization

**Figure 1:**
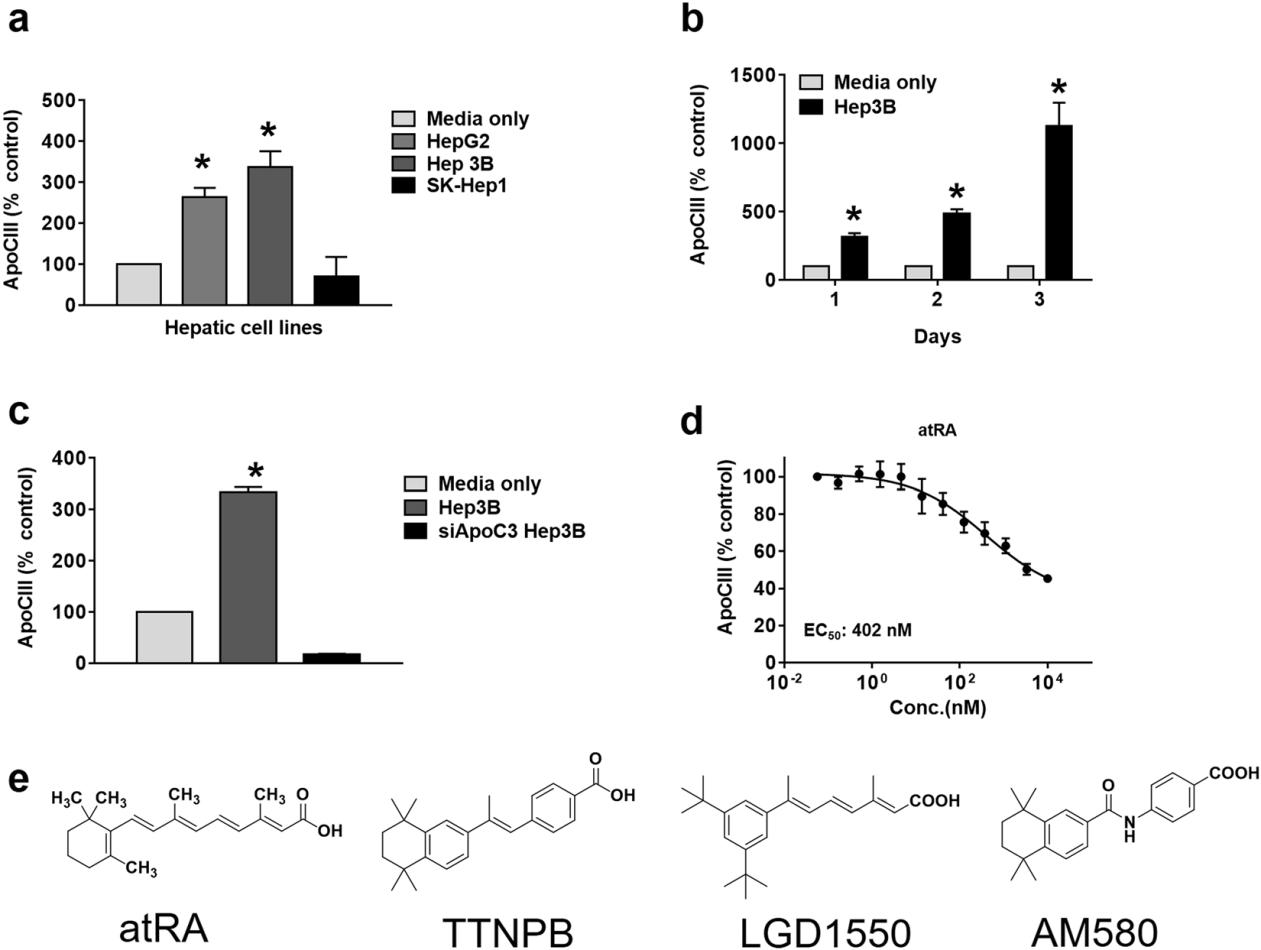
ApoC-III assay development and high throughput screening. (**a**) Secreted ApoC-III levels were measured in various hepatic cell lines (HepG2, Hep3B and SK-Hep1) after 3 days of incubation using an ApoC-III HTRF assay. Media only (no cells) readings represent background counts and were subtracted from all the raw data. (**b**) Hep3B cells were cultured for differing periods of time and secreted ApoC-III levels were measured by HTRF assay. (**c**) Hep3B cells were transfected with the siApoC-III as a positive control. (**d**) atRA was identified as a potential ApoC-III inhibitor in the primary screen. (**e**) Chemical structures of the RAR agonists, atRA, TTNPB, LGD1550 and AM580. Representative triplicate data is shown from at least three independent experiments.

A homogenous time-resolved fluorescence (HTRF) assay for ApoC-III (CISBIO, Codolet, France) was optimized to detect secreted ApoC-III levels in a cell-based system in a 1536-well plate format. Different human hepatic cell lines were tested and Hep3B cells were shown to secrete the highest level of ApoC-III in Minimum Essential Medium (MEM, Life Technologies, Carlsbad, CA) and 10% FBS, with a three-day incubation period (Fig. 1a,b). As there are no known small molecule ApoC-III inhibitors, we used siRNA to silence ApoC-III gene expression (~80% reduction) as a positive control for high throughput screening (Fig. 1c). A pilot screen validated the screening assay with a robust Z score (Z’ > 0.6) and a slight variation (CV < 5%).

We next carried out a high throughput screen (HTS) of a library of more than 950,000 small molecules to identify small molecule ApoC-III inhibitors. Hits were defined as compounds that inhibited >50% of ApoC-III secretion in the primary screen. Confirmed hits (>50% inhibition in two out of the three replicates) were further tested for dose-response (8 doses in 1:3 serial dilutions) in ApoC-III and cell viability assays, starting from 10 μM. Luminescence-based total ATP detection by Cell Titer-Glo (CTG) (Promega, Madison, WI) was used for the cell viability/cellular toxicity assay (Fig. S1). Among all the hits from the screen, we found that all *trans*-retinoic acid (atRA) potently blocked ApoC-III secretion up to 55% in culture media (*EC*_50_ = 402 nM) (Fig. 1d), and therefore chose to characterize this molecule further.

**Figure 2:**
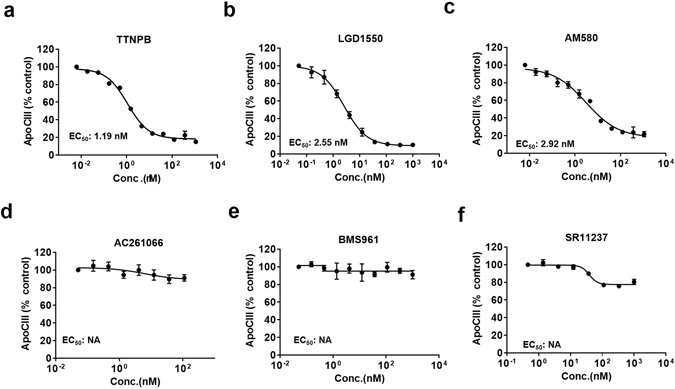
Effect of specific RARα activation on ApoC-III secretion. Hep3B cells were stimulated with the indicated RAR agonist for 3 days and secreted ApoC-III protein levels were measured by an ApoC-III HTRF assay: (**a**) TTNPB, RAR pan-agonist. (**b**) LGD1550, RAR pan-agonist. (**c**) AM580, RARα agonist. (**d**) AC21066, RARβ agonist. (**e**) BMS961, RARγ agonist. (**f**) SR11237, RXR agonist. Representative triplicate data is shown from at least three independent experiments.

The effects of retinoids are mediated by two families of nuclear retinoid receptors, the RARs and the retinoid X receptors (RXRs). Therefore, we first sought to determine the nuclear hormone receptor that is mediating ApoC-III secretion, using pharmacological nuclear receptor agonists. TTNPB and LGD1550, two known high-affinity ligands for RARs^26, 27^, inhibited secreted ApoC-III levels up to 90%, respectively, after incubation with Hep3B cells for 72 hours; TTNPB has an *EC*_50_ of 1.19 nM and LGD1550 has an *EC*_50_ of 2.55 nM, (Fig. 2a and b). This reduction of ApoC-III secretion was not caused by cellular toxicity, as indicated by lack of changes in total ATP levels that correspond cell viability (Fig. S1). In contrast, the selective RXR agonist SR11237 did not reduce ApoC-III secretion (Fig. 2f), suggesting that the ApoC-III effects are mediated by RAR. Although previous study reported that cis- retinoic acid increased ApoC-III levels potentially though activation of the RXR homodimers^28^. We did not observe a significant change in the presence of RXR agonists, potentially due to the differences in hepatic cell lines used.

### Specific activation of RARα inhibited ApoC3 production and secretion

**Figure 3:**
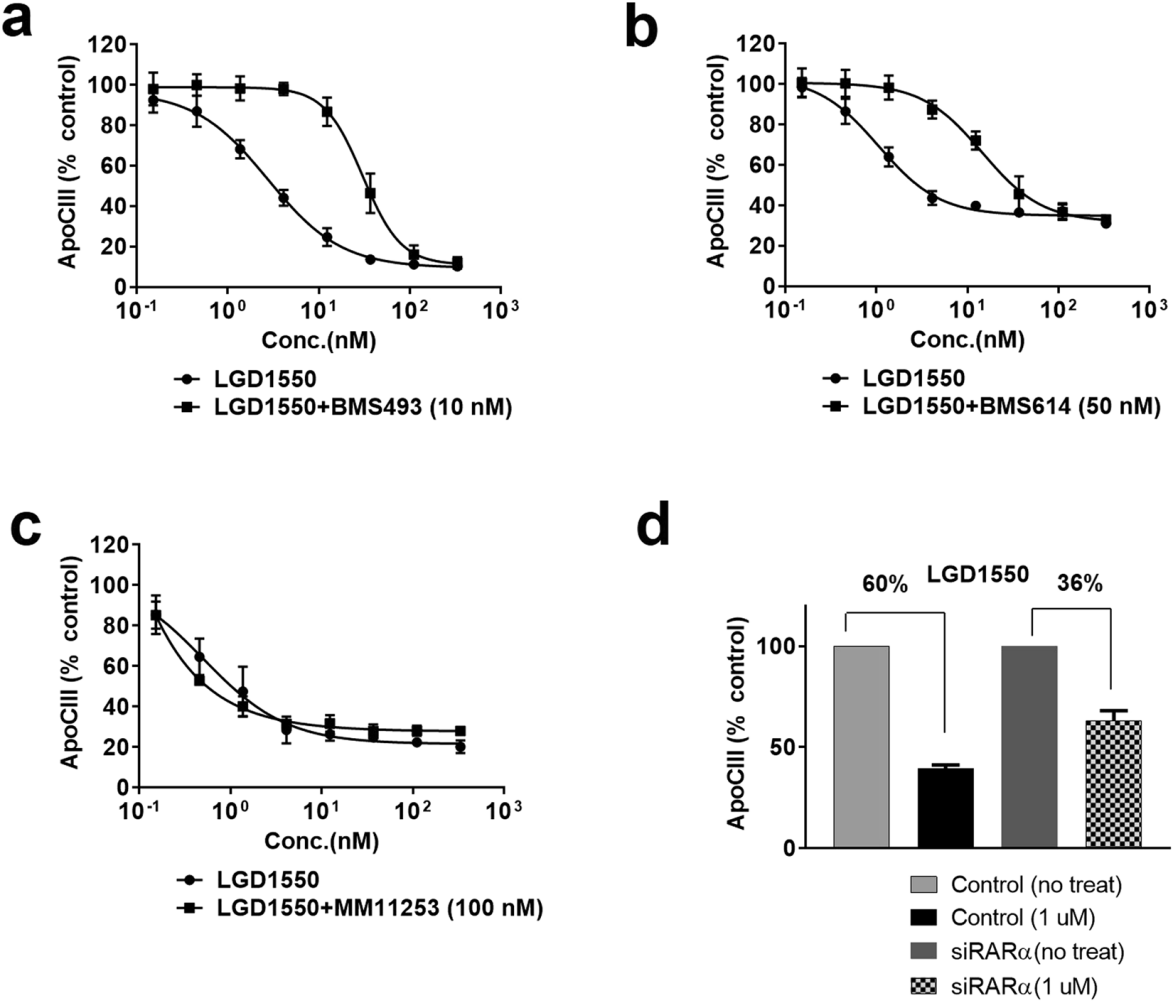
RARα antagonists reduce ApoC-III inhibition by RAR agonist. Hep3B cells were pretreated with various RARα antagonists 2 hrs before RAR agonist stimulation; cells were then incubated for 3 days and secreted ApoC-III protein levels were measured using the ApoC-III HTRF assay. (**a**) LGD1550 co-treated with 10 nM of BMS493, an RAR pan antagonist. (**b**) LGD1550 co-treated with 50 nM of BMS614, an RARα specific antagonist. (**c**) LGD1550 with 100 nM of MM11253, a specific RARγ agonist. (**d**) RARα in Hep3B cells were silenced by transient transfection with siRNA. Cells with or without siRARα were stimulated with 1 μM of LGD1550 and ApoC-III levels were measured after 3-day incubation. Representative triplicate data is shown from at least three independent experiments.

Since TTNPB and LGD1550 are non-specific RARα, β and γ agonists (termed pan-agonists), we sought to further delineate which specific RARs may be involved in ApoC-III inhibition. Various synthetic RARα, β, and γ specific agonists were tested in the ApoC-III secretion assay. The RARα specific agonist AM580 reduces ApoC-III secretion by 80% (*EC*_50_ = 2.92 nM; Fig. 2c), however, neither the RARβ agonist AC261066 nor the RARγ agonist BMS961 affected ApoC-III secretion (Fig. 2d and e). Consistent with this result, adenoviral overexpression of RARα also reduced ApoC-III secretion, whereas adenoviral overexpression of RARβ had no effect (Fig. S2). Cell toxicity was not observed under any of the above treatment conditions (Fig. S2). Furthermore, ApoC-III inhibition induced by pan-RAR agonists can be abolished by a pan-RAR antagonist and a RARα specific antagonist, but not by an RARγ specific antagonist (Figs 3 and S3). Specifically, exposure of Hep3B cells simultaneously to the RAR pan-agonist LGD1550 and the RAR pan-antagonist BMS493 diminished the inhibitory effect displayed by the agonist (Fig. 3a). Similarly, BMS614, a specific RARα antagonist, also diminished the inhibitory effect of LGD1550, similarly to the RAR pan-antagonist (Fig. 3b). In contrast, the RARγ specific antagonist MM11253 had no effect in similar experiments (Fig. 3c). Furthermore, gene-silencing of RARα using a pool of specific siRNAs in cultured Hep3B cells resulted in 80% knockdown of RARα (Fig. S3) which diminished the inhibitory effect of LGD1550 on ApoC-III secretion by 50% (Fig. 3d). These observations were also confirmed with another pan-RAR agonist TTNPB (Fig. S3). Taken together, these observations indicate that the reduction of ApoC-III secretion is selectively mediated via RARαactivation.

### RARα agonist reduced fatty liver in high fat diet (HFD) mouse model

**Figure 4:**
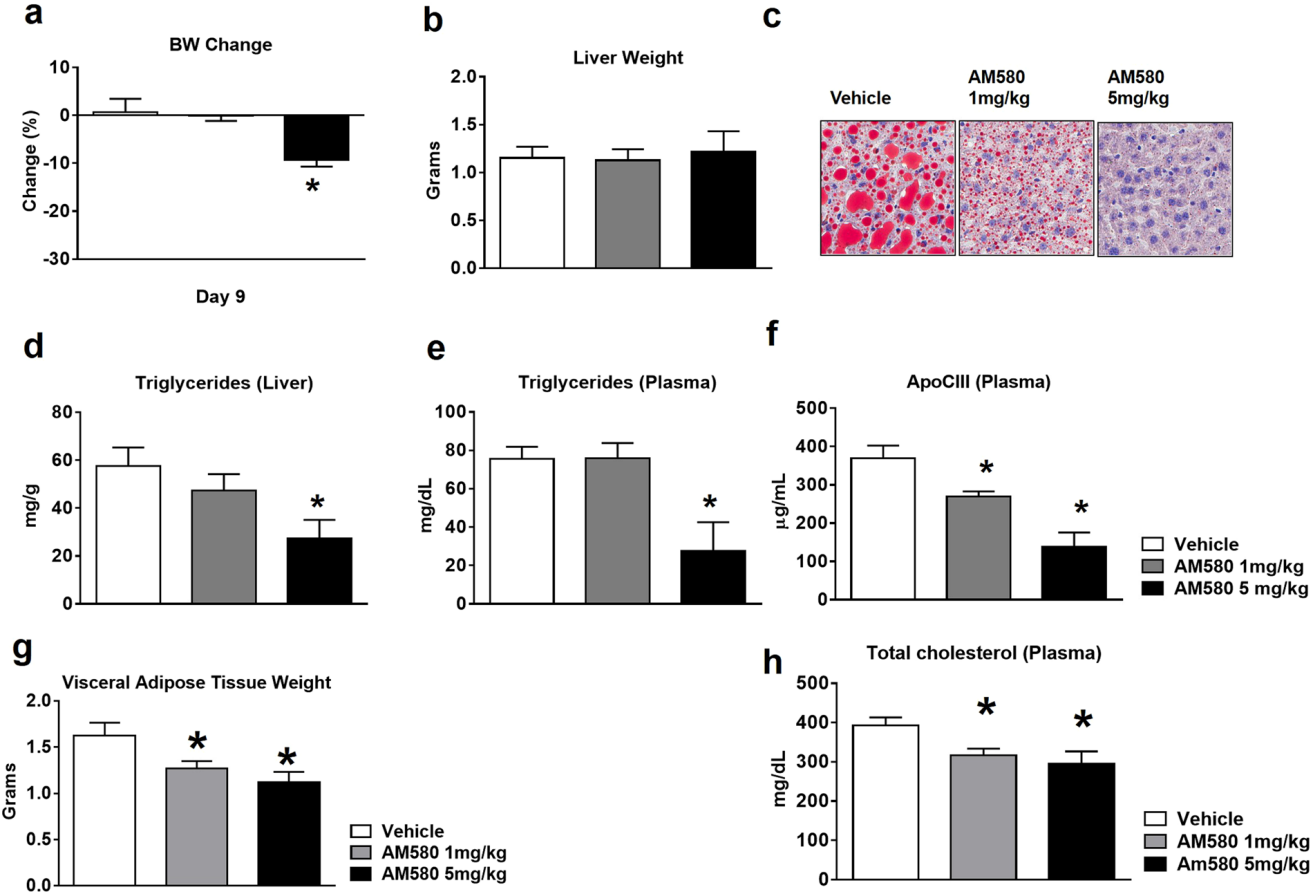
Effect of RARα agonist on ApoC-III and TGs production in high fat diet induced fatty liver mice. 4-month-old HFD mice treated daily with AM580 at 1 and 5 mg/kg by oral gavage. After 9 days, mice were sacrificed for plasma and tissue level analysis of ApoC-III and TGs and TCs. (**a**) Percentage body weight change; (**b**) Liver weight; (**c**) Hepatic lipids were stained by oil red O staining; (**d**) TGs levels in liver lysate and (**e**) Plasma TGs levels. (**f**) Plasma ApoC-III levels were measured by ELISA assay; (**g**) Visceral adipose tissue weights and (**h**) Plasma total cholesterol levels were measured by biochemical assay after 9 days of AM580 treatment in DIO mice. Data are means ± S.E. of 5–8 animals per group. *P < 0.05 versus vehicle.

To determine the *in vivo* activity of this novel mechanism, we evaluated the effects of the RARα agonist AM580 in the high fat diet induced fatty liver mouse model. First, we performed a pharmacokinetic study with AM580. Oral dosing AM580 (20 mg/kg) in mouse affords a *t*_1/2_~1.4 hrs, *C*_Max_~7026 ng/mL, and AUC ~12519 hr*ng/mL (n = 3) (Fig. S4). Based on these results, we administered AM580 (1 and 5 mg/kg doses) and vehicle daily by oral gavage to 4-month-old HFD mice for 9 days. AM580 in doses of 5 mg/kg reduced body weight by 10% (Fig. 4a). The weight of visceral adipose tissue decreased significantly (Fig. 4g), without affecting the liver weight (Fig. 4b). Histopathologic evaluation revealed AM580 treatment significantly reduced hepatic lipid accumulation as indicated by standard oil red O staining (Fig. 4c). Consistent with reduction of hepatic lipid accumulation, hepatic and plasma TGs, as well as plasma TC levels, were markedly reduced at 1 and 5 mg/kg of AM580 (Fig. 4d,e and h). Notably, serum ApoC-III levels were also significantly reduced after AM580 treatment (Fig. 4f). These findings indicate that inhibition of hepatic ApoC-III levels by activation of RARα leads to a reduction of serum TG levels and serum TC levels, and a corresponding reduction in hepatic lipid accumulation and body weight.

### RARα agonist reduces hepatic ApoC-III gene expression via reduction of HNF4α levels

**Figure 5:**
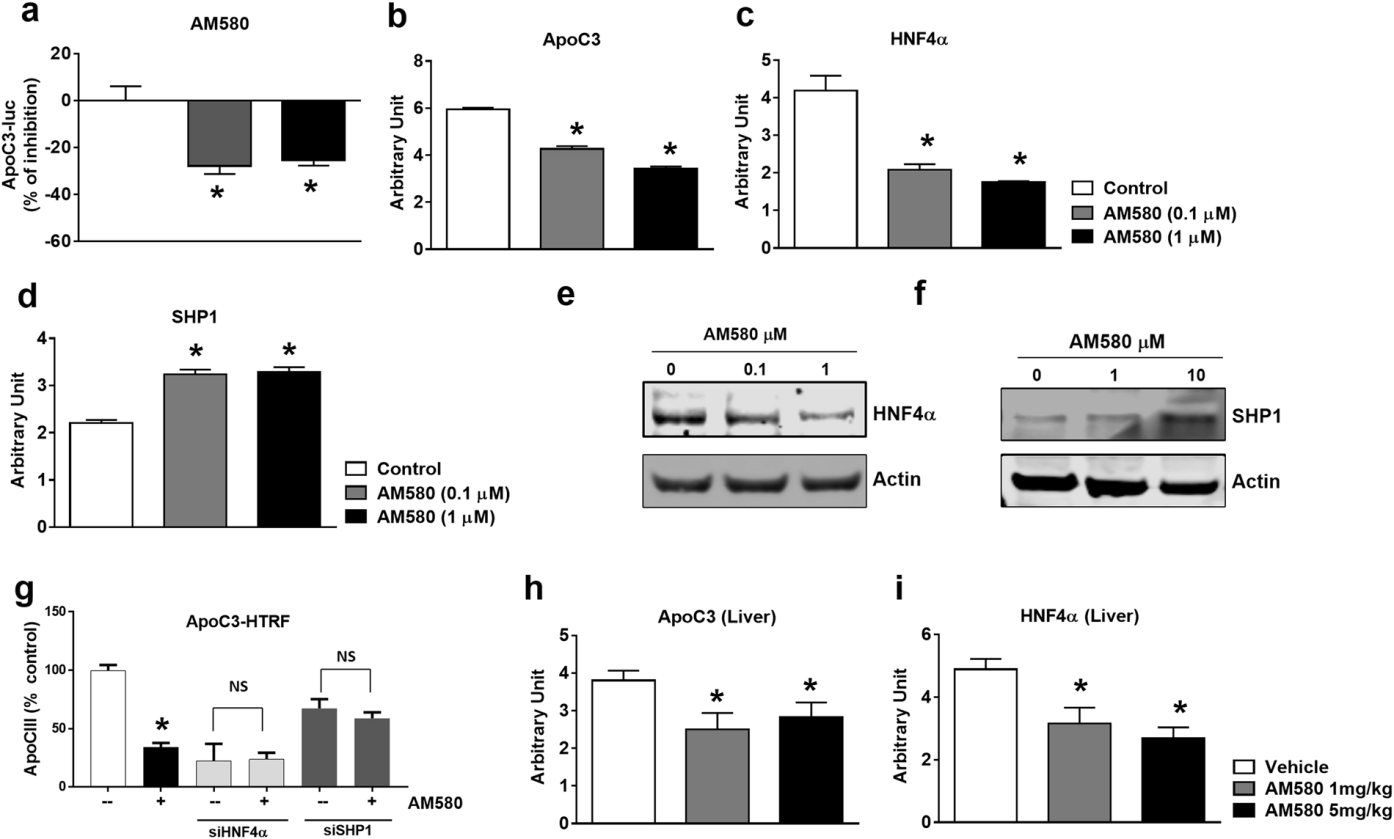
RARα agonist inhibits hepatic ApoC-III expression. (**a**) ApoC-III promoter activities were measured with an ApoC-III luciferase reporter assay in HepG2 cells. ApoC-III promoter reporter stable cells were generated by transfection and selection. ApoC-III luciferase reporter activities were measured after 16 hr incubation with AM580 (0.1 and 1 μM). mRNA levels of ApoC-III (**b**), HNF4α (**c**) and SHP1 (**d**) and protein levels of HNF4α (**e**) and SHP1 (**f**) in Hep3B cells. mRNA was analyzed by qRT-PCR in Hep3B cells after 16 hr incubation with AM580 (0.1 and 1 µM) or vehicle control. Protein levels were analyzed by western blotting with anti-HNF4α and anti-SHP1 antibodies after Hep3B cells were treated with Am580 (0.1, 1 or 10 μM) for 24 h. (**g**) ApoC-III protein levels are regulated by HNF4α-SHP1 axis. Hep3B cells were silenced with siHNF4α or siSHP1 in the presence or absence of AM580 (10 μM) and secreted ApoC-III levels at the end of day 3 were measured by HTRF assay as described earlier. Representative triplicate data is shown from at least three independent experiments. Liver mRNA levels of ApoC-III (**h**) and HNF4α (**i**) after 9 days of treatment with AM580 (1 mg/kg and 5 mg/kg) and vehicle control. Hepatic gene expressions were measured by qRT-PCR in liver tissue after 9 days of treatment. *P < 0.05 versus control. Data are means ± S.E. of 5–8 animals per group. *P < 0.05 versus vehicle.

To determine whether RARα agonists alter ApoC-III transcriptional activity, we developed an ApoC-III luciferase gene reporter assay in HepG2 cells. AM580 decreases ApoC-III transcriptional activity in the reporter assay (Fig. 5a); consistent with this result, the mRNA levels of ApoC-III were also significantly reduced by AM580 in Hep3B cells (Fig. 5b). These findings suggest that RARα agonist regulates ApoC-III gene expression, rather than secretion. In previously published work, a number of novel promoters of ApoC-III were reported, including MDM2 and HNF4α^29–31^. Aslo, *all-trans*-RA can downregulate α-fetoprotein (AFP) in Hep3B cells, potentially through HNF1α and HNF4α, although the detailed regulation mechanism is not fully elucidated^32^. SHP1, one of the orphan nuclear hormone receptors, interacts with a number of other nuclear hormone receptors, including HNF4α, and regulates their transcriptional activities^33^. We looked at the levels of HNF4α and SHP1 in Hep3B cells and noticed that HNF4α expression was significantly reduced, while SHP1 was significantly upregulated in the presence of AM580 (Fig. 5c and d, respectively). The same trend was observed in the protein levels of HNF4α and SHP1 in Hep3B cells treated with AM580 (Fig. 5e and f). Like AM580, the pan-agonists TTNPB and LGD1550 also inhibited ApoC-III transcription in the reporter gene assay (Fig. S6) and mRNA levels by qPCR (Fig. S6) in Hep3B cells, likely through SHP1 upregulation and HNF4α downregulation (Fig. S7).

To further confirm the role of HNF4α and SHP-1 in regulating ApoC-III, we silenced HNF4α and SHP1 using siRNAs. To our satisfaction, silencing of HNF4α with siRNA phenocopies AM580 treatment wherein ApoC-III mRNA and protein levels were reduced while SHP1 mRNA levels are increased (Fig. S8). On the other hand, silencing of SHP1 increased ApoC-III mRNA and protein levels while increasing HNF4α mRNA levels (Fig. S8). Furthermore, silencing of *HNF4α* in the presence of AM580 does not further reduce the ApoC3 protein levels, while silencing of SHP1 abolished the effect of AM580 on the ApoC3 protein levels (Fig. 5g). Consistent with these *in vitro* observations, reduced expression of ApoC-III and HNF4α were confirmed in liver after 9 days of AM580 treatment (Fig. 5h and i). Taken together, these findings strongly suggest that RARα agonists modulate hepatic ApoC-III synthesis through regulation of SHP1 and HNF4α transcriptional regulators.

## Discussion

Several rodent and human genetic studies have validated a central role for ApoC-III in triglyceride homeostasis. In addition, recent epidemiological and population-based studies have linked elevated plasma ApoC-III levels with hypertriglyceridemia, risk of cardiovascular disease and metabolic syndrome^3–10, 18^. To identify novel small molecules that directly target ApoC-III production, processing and secretion, we carried out a large-scale, cell-based screen and identified RARα agonists as novel small molecule ApoC3 inhibitors. RA related receptors consist of RARs (RARα, β, and γ) and RXRs (RXRα, β, and γ)^34, 35^. The RAR/RXR heterodimers bind to the polymorphic *cis*-acting response elements of RA gene targets and exert a variety of effects. RA plays a critical role in normal development, growth, and maintenance of certain tissue^36–38^. In addition, RA plays a significant role in lipid metabolism. RARα-dominant negative transgenic mice develop steatohepatitis and subsequent hepatic malignancies^39^ and the observed pathology can be reversed by an RA-enriched diet. In addition, hepatic retinoid signaling is impaired in humans with NASH and RARα2 genes were significantly decreased in NASH, compared to that in age-matched controls^40^. These studies suggest that hepatic loss of RA function may contribute to impaired hepatic lipid metabolism and subsequently to NASH.

We have identified a RARα specific role in the modulation of ApoC-III levels. *In vitro* experiments indicate that specific activation of RARα inhibits hepatic mRNA and protein level of ApoC-III in Hep3B cells. Subsequent *in vivo* experiments with DIO mice corroborated these findings and showed a significant reduction in total body weight and plasma TG levels. Consistent with these observations, plasma and hepatic ApoC-III levels were also markedly reduced in these experiments.

We observed similar efficacy with atRA treated mice (Fig. S5), which has been previously reported to significantly reduce hepatic lipid accumulation and blood lipid levels (TGs and TCs) in DIO mice^41^. Unfortunately, atRA increases TG levels in acute promyelocytic leukemia patients^42, 43^. The reasons for this discrepancy are not clear, but are likely to involve the RARβ or γ activation and dimerization with RXR^44, 45^. In addition, this finding may be specific to APL as a disease entity and not reflective of the effects that may occur in a healthy patient without hematologic malignancy. Moreover, RARβ and γ are associated with unexpected side effects^44^. Therefore, downregulation of ApoC-III by selective RARα activation may lead to multiple beneficial effects in terms of metabolic disease without the unwanted side effects.

Several pathways have been proposed for the regulation of ApoC-III gene expression as well as transport to the blood stream. ApoC-III gene expression is regulated, in part, by the insulin-response element (IRE) on the ApoC-III gene^46^ and ApoC-III is downregulated by insulin^47^. Also, ApoC-III promoter regulation is regulated by multiple nuclear receptors and involves complex interactions between the receptors^48^. ApoC-III gene expression is also modulated by PPARs (peroxisome-proliferator-activated receptors) causing a reduction in levels with induction of PPARα^30, 31, 49^. Several therapeutic agents indirectly regulate ApoC-III expression through activation of PPARα and reduce plasma ApoC-III levels in metabolic syndrome^50^. PGC-1β and HNF4α also regulate plasma triglyceride metabolism by stimulating ApoC-III expression and elevating ApoC-III levels in circulation^51^. In this work, we demonstrate that RARα inhibits hepatic ApoC-III expression through the SHP1-HNF4α axis. RARα increases SHP1 levels, a negative regulator of HNF4α, which results in decreased levels of ApoC-III gene expression. Furthermore, silencing of HNF4α phenocopies AM580 treatment while silencing of SHP1 has the opposite effect on ApoC-III. This mechanism was translated into high fat diet induced fatty liver models where treatment of AM580 resulted in reduced hepatic and secreted lipid levels, *potentially through on-target effect of increased lipolysis* ^11^. This potentially led to the reduced fat accumulation in adipose tissue. However, the exact mechanism of lipid movement and its effect on food intake, energy expenditure and body weight will warrant further detailed mechanistic studies in the future. Thus, we have identified a pathway that links the ApoC-III gene cluster to ApoC-III plasma and liver levels and can be modulated with selective RARα agonists. Considering the importance of targeting ApoC-III regulation in CVD, specific inhibition of ApoC-III through RARα activation may be a new therapeutic approach and specific RARα agonist AM580 may provide a lead compound for identification and development of more optimized agonists of RARα.

## Materials and Methods

### Cell culture, silence and overexpression

Hep3B cells were grown in MEM with 10% FBS at 37 °C in an atmosphere of 95% air −5% CO_2_. For silencing ApoC-III and RARα, commercial siRNA plasmids targeting human ApoC-III and RARα were obtained from Santa Cruz Biotechnology (Dallas, Texas). Hep3B cells were transiently transfected with siRNA using Lipofectamine (Life Technologies, Carlsbad, CA) following the protocols provided by the manufacturer. For overexpression of RARα, RARβ, Hep3B cells were infected with adenovirus for RARα, RARβ and GFP control from Vector Biolabs (Malvern, PA) following the protocols provided by the manufacturer.

### ApoC-III HTRF uHTS

The Hep3B cell line was the most responsive for ApoC-III secretion and a three-day incubation time after compound transfer gave us the maximum signal to noise in the 1536-well format, Hep3B cells (5 μl 500 cells/well) were first dispensed to each well of a white solid-bottomed 1536-well plate using a bottle valve liquid dispenser. After overnight incubation, cells were treated with 20 nL/well compound in 1 mM DMSO stock concentration (final concentration is 4 μM) or DMSO alone (neutral control) using a 1536-well head pintool unit. Plates were then incubated at 37 °C, 5% CO_2_ and 95% relative humidity. Three days later, plates were equilibrated to room temperature for 20 min and the HTRF assay was initiated by adding 5 μl of HTRF ApoC-III reagent to each well. After 2 hours of incubation, light emission was measured for 30 seconds with Envision multilabel plate readers (PerkinElmer). The ApoC-III levels were normalized to the neutral control (DMSO, 100%) on a per-plate basis, based on the ratio of A_665 nm_/A_615 nm_. Media only blank readout was subtracted from all the values before normalizing the data to the neutral control.

### Real-time PCR

Total RNA was extracted from frozen cell pellets using TRIzol reagent (Life Technologies, Carlsbad, CA). Tissue samples were homogenized and RNA extracted using RNeasy Mini Kits (Qiagen, Hilden, Germany) following the manufacturer’s protocol. The extracted RNA was treated with DNAase (Life Technologies, Carlsbad, CA) and reverse transcription (Qiagen, Hilden, Germany) was performed with 1 μg of total RNA. Quantitative real-time PCR was utilized to evaluate expression levels from the cDNA by using Absolute SYBR Green real-time PCR mastermix (Thermo Fisher Scientific, Waltham, MA) and a real-time PCR thermocycler (Effendorf, Hamberger, Germany). Expression levels of each gene were measured and then normalized with 18 S ribosome RNA.

### Animal maintenance and treatment

C57BL/6 J mice were placed on high fat diet (Research diet, D12492 containing 60% of calories from fat) at 6 weeks of age to induce obesity and fatty liver. 16-week-old high fat diet induced obese (DIO) mice were obtained from the Jackson Laboratory (JAX380050, Bar Harbor, ME). All animals were maintained under conditions of controlled temperature (22.5 °C) and illumination (12-h dark/light cycle). All procedures were approved by the Institutional Animal Care and Utilization Committees (IACUC) at California Institute for Biomedical research (Calibr), in accordance with the NIH guidelines for the care and use of laboratory animals. Mice were then treated once daily with vehicle (corn oil) or RARα agonist (AM 580) by oral gavage for 9 days with the high fat diet. At the end of the study, mice were sacrificed and plasma lipid profiles and liver fat contents were analyzed.

### Levels of plasma ApoC-III, TG and TC

For quantification of plasma ApoC-III levels, an Apo C-III rat/mouse ELISA Kit (Abnova, Taipei city, Taiwan) was used. Plasma and tissue triglyceride levels were measured using a Triglyceride Colorimetric Assay Kit (Cayman chemical, Ann Arbor, Michigan). Plasma total cholesterol was measured using a Cholesterol Assay Kit (Abcam, Cambridge, MA). All assays were performed per the manufacturer’s instructions.

### Statistics

Experiments were performed in triplicates in at least three independent experiments. *In vivo* data are means ± S.E. of 5–8 animals per group. Data were analyzed with StatView software (Abacus, Baltimore, MD) using one-factor analysis-of-variance analysis. P values less than 0.05 were considered statistically significant.

## Electronic supplementary material

Supplementary Information
