# Supplementary material for: A Novel Role for RARα Agonists as Apolipoprotein CIII Inhibitors Identified from High Throughput Screening
Source: Sci Rep. 2017 Jul 19;7:5824. doi: 10.1038/s41598-017-05163-w (PMC5517646; doi:10.1038/s41598-017-05163-w)
Supplement: Supplementary file 1 — Supplementary Information [file 41598_2017_5163_MOESM1_ESM.pdf]

Supplementary Material for

A Novel Role for RARα Agonists as Apolipoprotein CIII Inhibitors Identified from High Throughput Screening

Sang Jun Lee[1], Madhupriya Mahankali[1], Abdallah Bitar[2,3], Huafei Zou[1], Elizabeth Chao[1], Hung Nguyen[1], Jose Gonzalez[1], Dawna Caballero[1], Mitch Hull[1], Danling Wang[1], Peter G Schultz[1,2], and Weijun Shen[1,*]

[1]*California Institute for Biomedical Research (**Calibr**), La Jolla, CA 92307, USA*

[2]*Department of Chemistry, the Scripps Research Institute, La Jolla, CA 92037, USA*

[3]*Scripps Translational Science Institute, La Jolla, CA 92037, USA*

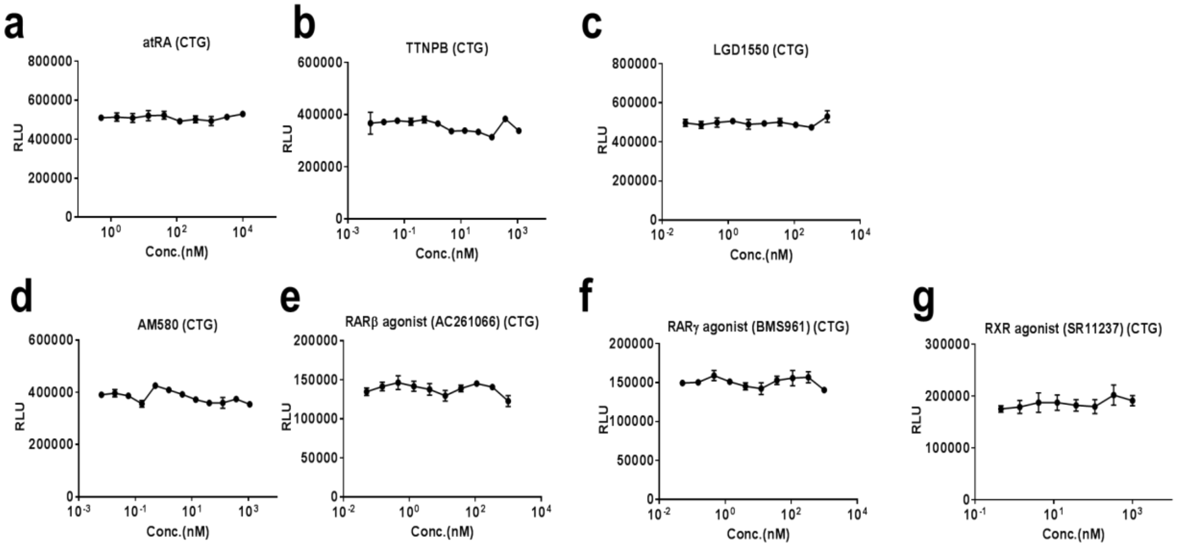

Supplementary Figure 1. Cell toxicity assay. Cell toxicities were measured after 16hr treatment with various RARα agonists using Luminescent Cell Viability Assay (CellTiter-Glo, Promega). (a) atRA; (b) TTNPB; (c) LGD1550; (d) Am580; (e) AC261066; (f) BMS961; (g) SR11237.

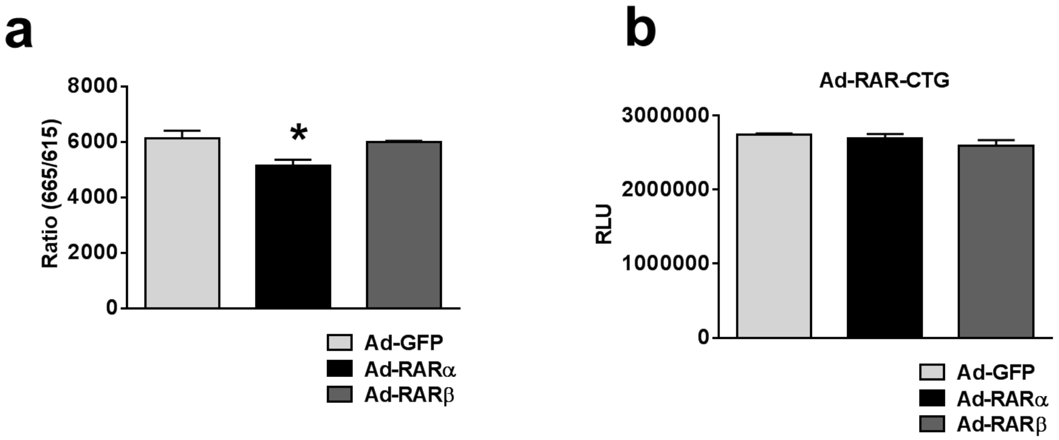

Supplementary Figure 2. Effect of RARα overexpression by adenovirus infection on ApoC-III. (A) RARα, RARβ and GFP alone were overexpressed in Hep3B cells by adenovirus infection for 16 hr. (A) RARα or β overexpressed Hep3B cells were incubated for 3 days and secreted ApoCIII levels were measured by ApoCIII HTRF assay. (B) Cellular toxicities were measured after 3 days of culture after adenovirus infection.

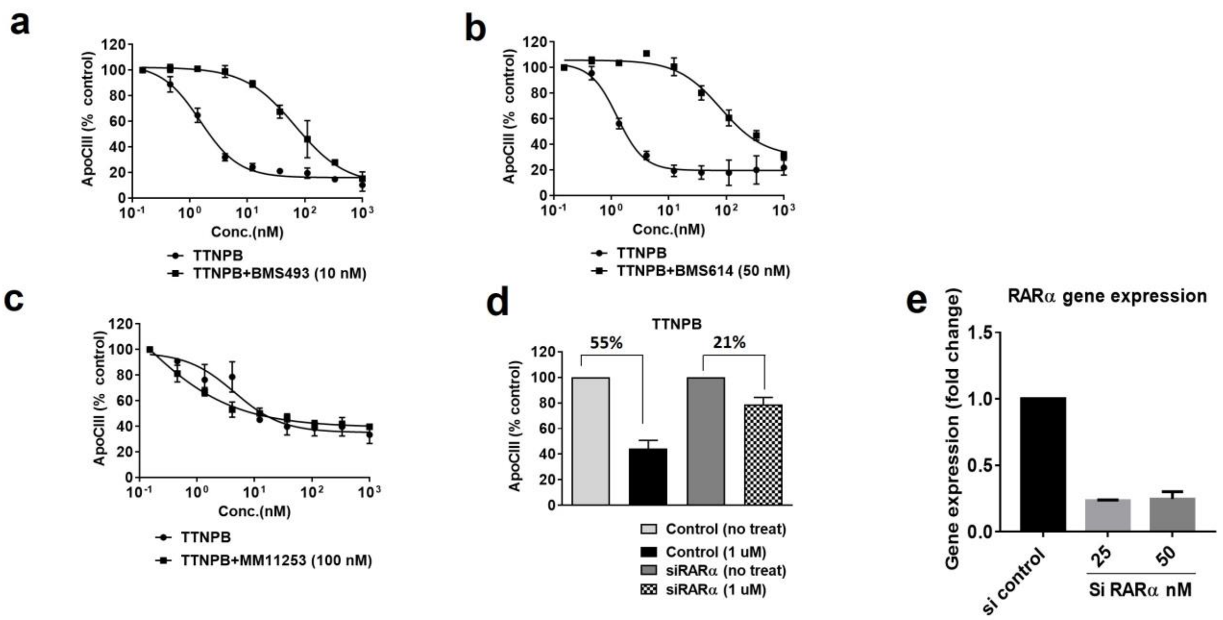

Supplementary Figure 3 RARα antagonists diminish ApoC-III inhibition by an RAR agonist. Hep3B cells were pretreated with various RARα antagonists 2h before RAR agonist stimulation. Cells were incubated for 72 hours and secreted ApoC-III protein levels were measured by ApoC-III HTRF assay. (a) TTNPB co-treated with 10 nM of BMS493, an RAR pan-antagonist. (b) TTNPB co-treated with 50 nM of BMS614, RARα antagonist. (c) TTNPB co-treated with 100 nM of MM11253, an RARγ agonist. (d) Hep3B cells were silenced with siRARα by transient transfection. Cells with or without RARα silencing were stimulated with 1 μM of TTNPB and ApoC-III levels were measured after 72 hours of incubation. (e) The extent of RARα knockdown is shown by qPCR analysis of RARα levels.

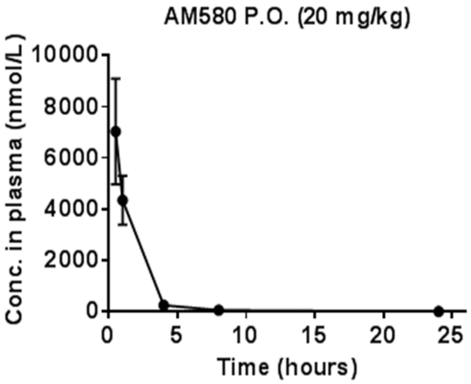

	t1/2	Cmax	AUC
	(hr)	(ng/mL)	(hr*ng/mL)
Mean	1.41256667	7026.6667	12519.0867
SD	0.64073667	1683.4554	1963.21196
%CV	0.45359747	0.2395809	0.15681751

Supplementary Figure 4 AM580 pharmacokinetics in mouse RARa agonist AM580 was dosed at 20mg/kg oral gavage to the female CD1 mice (n =3) and blood was drawn at 30min, 1h, 4h, 8h and 24h and plasma concentration of AM580 was analyzed by Shimadzu LC 10ADvp coupled with API3000 mass spec and quantified by the standard calibration curve using AM580 in control mice plasma.

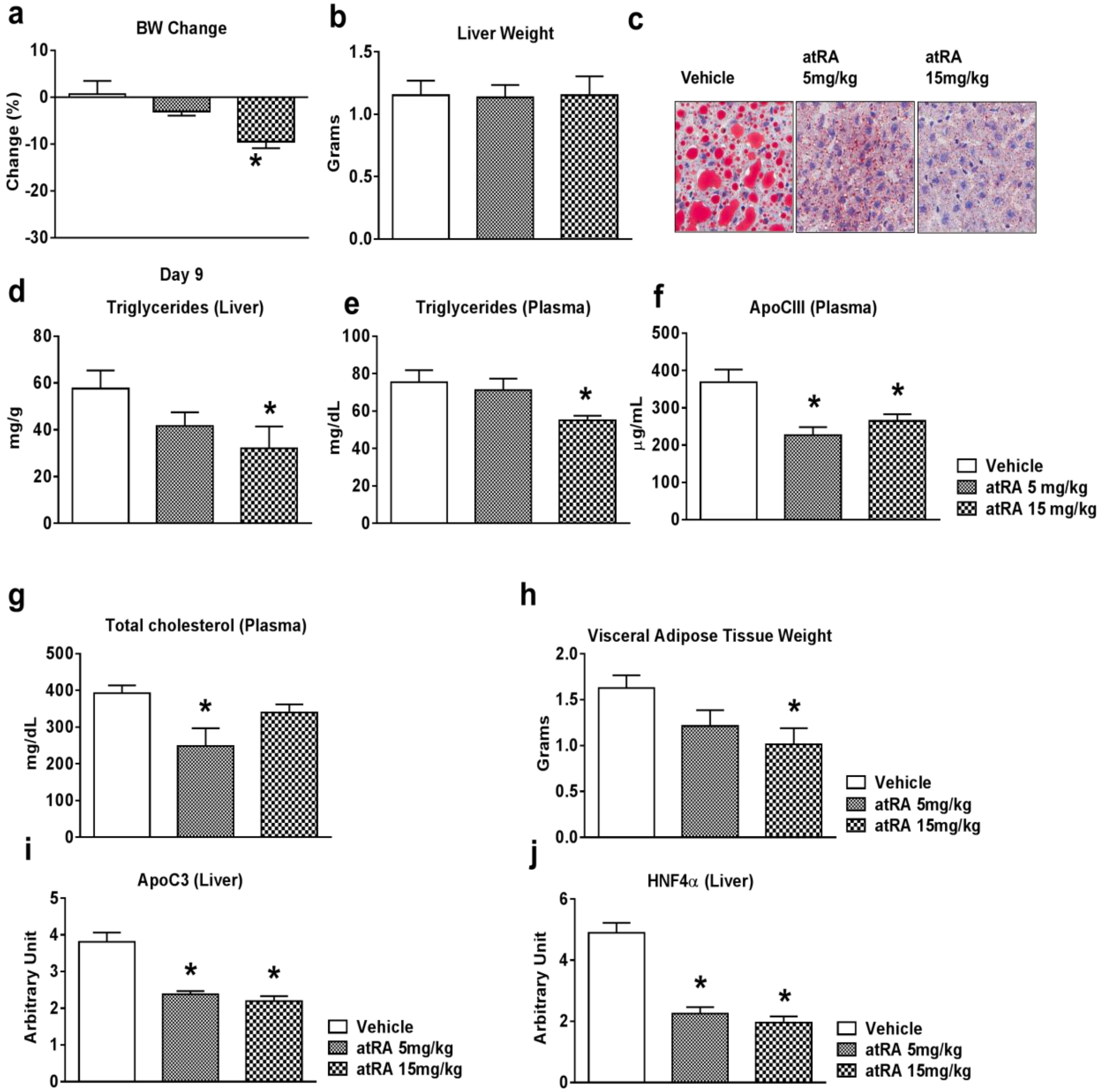

Supplementary Figure 5. Effect of RARα agonist on ApoC-III and TGs production in high fat diet induced fatty liver mice. 4 month-old HFD mice treated daily with atRA at 5 and 15 mg/kg by oral gavage. After 9 days, mice were sacrificed for plasma and tissue level analysis of ApoC-III and TGs and TCs. (a) Percentage body weight change; (b) Liver weight; (c) Hepatic lipids were stained by Oil red O staining; (d) Liver lysate TGs and (e) Plasma TGs levels. (f) Plasma ApoC-III levels were measured by ELISA assay; (g) total plasma cholesterol and (h) Visceral adipose tissue weights and Liver lysate mRNA levels of (i) ApoC-III (j) HNF4α were measured by biochemical assay after 9 days of atRA treatment in DIO mice. Data are means ± S.E. of 5-8 animals per group. *$P < 0.05$ versus WT.

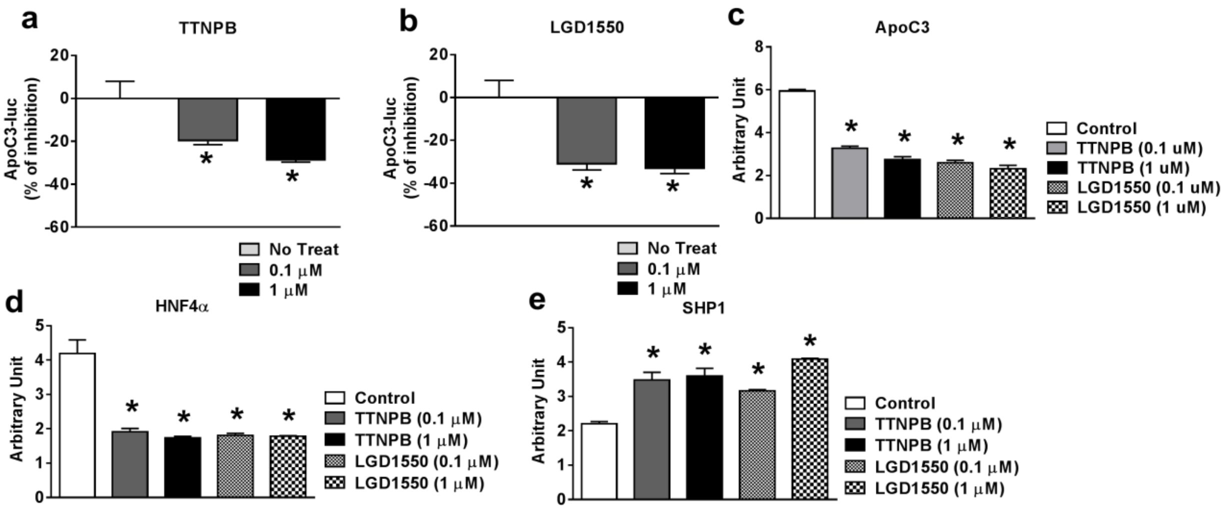

Supplementary Figure 6 RAR agonists inhibit hepatic ApoC-III gene expression by inhibition of HNF4α and upregulation of SHP1. ApoC-III promoter activities were measured by ApoC-III luciferase reporter assay in HepG2 cells. ApoC-III promoter reporter stable cells were generated by transfection and selection. ApoC-III luciferase reporter activities were measured after 16 hr stimulation of TTNPB and LGD1550 (0.1 and 1 μM) (a and b). mRNA was analyzed by qRT-PCR in Hep3B cells stimulated with TTNPB and LGD1550 (0.1 and 1 μM). (d) mRNA levels of ApoC-III. (d) mRNA levels of HNF4α. (e) mRNA levels of SHP1. Experiments were performed in triplicate and repeated at least twice. *$P < 0.05$ versus control.

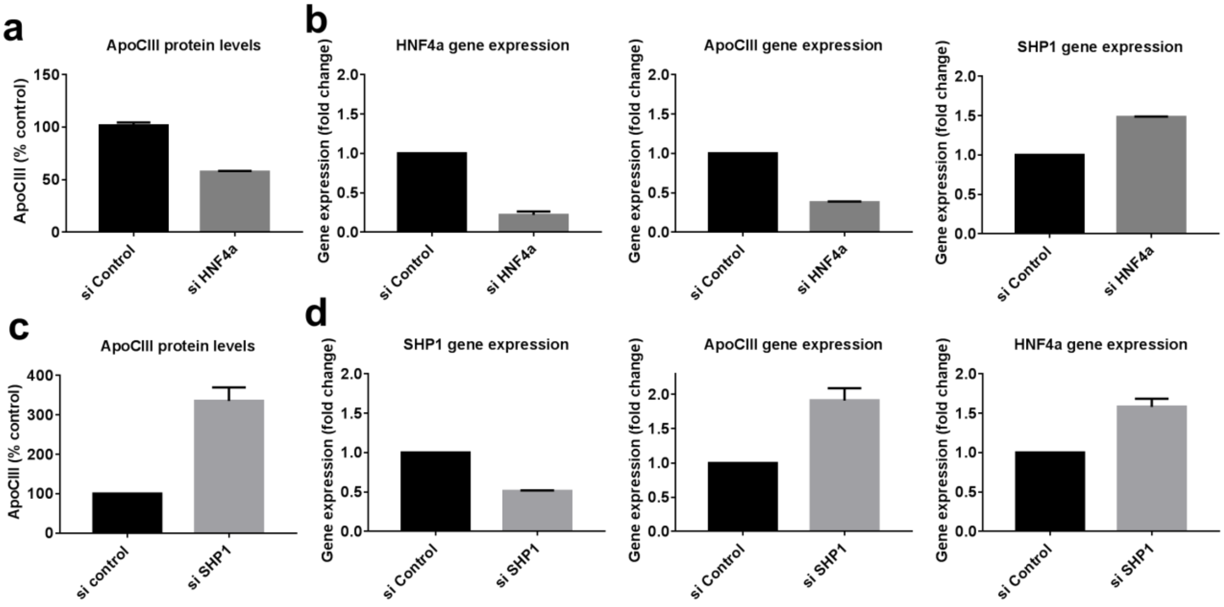

Supplementary Figure 7 ApoC-III protein and mRNA levels are regulated by HNF4α-SHP1 axis. Hep3B cells were silenced with siHNF4a or siSHP1 for 3 days. (a and c) Secreted ApoC-III levels were measured by HTRF assay as described earlier. (b and d) mRNA levels of HNF4a, SHP1 and ApoC-III were analyzed by qRT-PCR. Experiments were performed in triplicates.
